# Erythropoietin; a Novel Neuroprotective Agent for Ocular Disorders

**Published:** 2011-01

**Authors:** Khalil Ghasemi Falavarjani, Mehdi Modarres

**Affiliations:** Eye Research Center, Rassoul Akram Hospital, Tehran University of Medical Sciences

Neuroprotection aims to prevent neuronal injury, ideally with preservation of function. Many neurological disorders, including ischemic, traumatic, or degenerative insults, share common features of destruction of neurons mediated by similar pathophysiological mechanisms including apoptosis (programmed cell death), increased release of excitotoxic amino acids, (particularly glutamate), intracellular accumulation of calcium, oxidative stress by radical oxygen species, and inflammatory reactions accompanied by infiltration of cells and production of cytokines. It has been shown that neurons, like many other cells, have the potential to regenerate. The concept of pharmacologic neuroprotection is directed at halting or possibly reversing general mechanisms of cell death, especially apoptosis, and restoring or regenerating cell function rather than treating specific disease entities.

Erythropoietin (EPO), a 30.4 kilo Dalton glycoprotein hormone, promotes red blood cell differentiation by preventing apoptosis of erythroid progenitors in the bone marrow. In recent years, erythropoietin has been shown to possess potent neuroprotective and neuroregenerative properties. This agent has been shown to reduce retinal ganglion cell apoptosis *in vitro*, a finding which was later corroborated by *in vivo* studies. Retinal ganglion cell survival in animal models was promoted by erythropoietin after optic nerve transection, chronically elevated intraocular pressure and diabetic retinopathy.

Recently, promising results have been reported using systemic and intravitreal erythropoietin for different ocular conditions. Kashkouli et al evaluated the effect of intravenous erythropoietin for treatment of indirect traumatic optic neuropathy (TON) and compared the outcomes of such treatment with no specific intervention. Mean best-corrected visual acuity (BCVA) significantly (P=0.028) improved from 1.82 logMAR at baseline to 0.94 logMAR at final follow-up in the EPO group, whereas the observation group showed insignificant (P=0.28) visual improvement from 2.55 logMAR to 2.32 logMAR. Li et al determined the effect of intravitreal erythropoietin in 5 eyes with severe, chronic diabetic macular edema unresponsive to prior multi-modal treatment. They reported that visual acuity improved by 3 lines or more in 3 eyes and by 1 line in 2 eyes. Visual improvement occurred within a week after initial intravitreal EPO injection and was maintained up to 18 weeks. Modarres et al reported the results of intravitreal injection of erythropoietin in eyes with non-arteritic anterior ischemic optic neuropathy. After the injections, 64.5% and 54.8% of eyes experienced 3 or more lines of improvement in visual acuity at 3 months and at final follow-up, respectively. Their results were distinctly superior to the natural course of the disease (39.5% rate of improvement of 3 lines or more after 3 months according to the Ischemic Optic Neuropathy Decompression Trial).

Neuroprotection is an evolving strategy for limiting or possibly reversing neuronal/axonal injury due to a variety of insults. This concept may be used as a therapeutic option in glaucoma, vascular occlusions, or retinal degenerative disorders. Further studies are required to elucidate the potential benefits of erythropoietin in different ocular conditions.
